# Conformity of the NASADEM_HGT and ALOS AW3D30 DEM with the Altitude from the Brazilian Geodetic Reference Stations: A Case Study from Brazilian Cerrado

**DOI:** 10.3390/s21092935

**Published:** 2021-04-22

**Authors:** Giovana Maranhão Bettiol, Manuel Eduardo Ferreira, Luiz Pacheco Motta, Édipo Henrique Cremon, Edson Eyji Sano

**Affiliations:** 1 Image Processing and GIS Lab (LAPIG), Federal University of Goiás (UFG), Goiânia 74001-970, Brazil; manuel@ufg.br; 2 Embrapa Cerrados, Planaltina 73301-970, Brazil; edson.sano@embrapa.br; 3 Brazilian Institute of Environment and Renewable Natural Resources (IBAMA), Brasília 70818-900, Brazil; luiz.motta@ibama.gov.br; 4 Federal Institute of Education, Science and Technology of Goiás (IFG), Goiânia 74055-110, Brazil; edipo.cremon@ifg.edu.br

**Keywords:** digital elevation model, cartographic accuracy standard, Brazilian Geodetic System, PEC-PCD

## Abstract

The Brazilian Cerrado (tropical savanna) is the second largest biome in South America and the main region in the country for agricultural production. Altitude is crucial information for decision-makers and planners since it is directly related to temperature that conditions, for example, the climatic risk of rainfed crop plantations. This study analyzes the conformity of two freely available digital elevation models (DEMs), the NASADEM Merged Digital Elevation Model Global 1 arc second (NASADEM_HGT) version 1 and the Advanced Land Observing Satellite Global Digital Surface Model (ALOS AW3D30), version 3.1, with the altitudes provided by 1695 reference stations of the Brazilian Geodetic System. Both models were evaluated based on the parameters recommended in the Brazilian Cartographic Accuracy Standard for Digital Cartographic Products (PEC-PCD), which defines error tolerances according to eight different scales (from 1:1000 to 1:250,000) and classes A (most strict tolerance, for example, 0.17 m for 1:1000 scale), B, C, and D (least strict tolerance, for example, 50 m for 1:250,000 scale). Considering the class A, the NASADEM_HGT meets 1:250,000 and lower scales, while AW3D30 meets 1:100,000 and lower scales; for class B, NASADEM_HGT meets 1:100,000 scale and AW3D30 meets 1:50,000. AW3D30 presented lower values of root mean square error, standard deviation, and bias, indicating that it presents higher accuracy in relation to the NASADEM_HGT. Within eight of Cerrado’s municipalities with the highest grain production, the differences between average altitudes, measured by the Cohen’s effect size, were statistically insignificant. The results obtained by the PEC-PCD for the Cerrado biome indicate that both models can be employed in different DEM-dependent applications over this biome.

## 1. Introduction

Digital elevation models (DEMs) have been applied in different studies, including water management, geomorphology and landscape analysis, volcanic activity monitoring, and sea level change detection [1]. Grohmann [2] also pointed out other applications such as development of geopotential global models, evaluation of glacier volume change, climatic modeling, vegetation mapping, and development of navigation systems for commercial aviation. Most of the environmental and geological studies need accurate elevation data with global coverage [1]. The high demand for DEMs accelerated the launch of satellites that collect stereo pair data in the optical spectral range as well as interferometric synthetic aperture radar (InSAR) data in the microwave spectral range.

The most popular and freely available DEMs are the 1 arc second (~30-meter spatial resolution) Shuttle Radar Topography Mission (SRTM) [3] and the Advanced Spaceborne Thermal Emission and Reflection Radiometer (ASTER) Global Digital Elevation Model (GDEM) [4,5]. More recently, the German Aerospace Center (DLR) produced a global, consistent, and high-resolution (12-meter spatial resolution) DEM with unprecedented accuracy based on the TanDEM-X mission [6]. These data are only freely available for scientific and commercial purposes. Other DEMs are the 30-meter spatial resolution NASADEM, considered as the successor of SRTM, that was produced by reprocessing the SRTM and merging it with ASTER GDEM [7], as well as the Advanced Land Observing Satellite Global Digital Surface Model (ALOS AW3D30; 30-meter spatial resolution), produced based on the ALOS Panchromatic Remote Sensing Instrument for Stereo Mapping (PRISM) [8]. Recently, NASA made available the NASADEM Merged Digital Elevation Model Global 1 arc second (NASADEM_HGT) data layers that include the DEM itself, the number of scenes processed by each pixel, and an updated SRTM water body mask [9].

Several studies have been published assessing the vertical accuracy of these products. For example, the vertical accuracy of AW3D30 was assessed by [2,5,10], while NASADEM accuracy was assessed by [11,12]. Assessment of the accuracy of satellite-based DEMs is based on the comparison with the altitudes provided by high-precision global navigation satellite systems (GNSS) receivers, geodetic marks, and laser scanning data, among other products. For example, Gdulová et al. [13] assessed the vertical accuracy of TanDEM-X DEM (12-meter spatial resolution) over a European mountain environment based on the airborne LiDAR data, presenting vertical accuracy exceeding 30 cm. They found that this product complies with the 10-m mission specification benchmark: in non-forested areas, the LE90 (90% confidence of vertical accuracy) reached values below 6 m, while in coniferous forests, it was equal to or below 12 m. González-Morada and Viveen [14] compared ASTER GDEM, SRTM, AW3D30, and TanDEM-X DEMs against a set of 139 measurements gathered by a dual-frequency Trimble 5800 Global Navigation Satellite System (GNSS) receiver. The root mean square error (RMSE) were below 7 m for all models, especially for TanDEM-X (RMSE = 1.7 m).

In Brazil, the accuracy of DEMs is often presented in terms of Standard of Cartographic Accuracy of Digital Cartographic Products (PEC-PCD, abbreviation in Portuguese), according to the DEM’s RMSE, eight scales of evaluation (1:1000, 1:2000, 1:5000, 1:10,000, 1:25,000, 1:50,000, 1:100,000, and 1:250,000), and classes A, B, C, and D (see Section 2.1 and Section 2.2 for details). Viel et al. [15] evaluated the PEC-PCD of SRTM, AW3D30, and ASTER GDEM from a test site located in the Rio Grande do Sul State, Brazil. Results showed that all models fitted 1:50,000, class D. According to Barbosa et al. [16], AW3D30 DEM reached a PEC-PCD of 1:25,000, class C, in a study area located in the municipality of Formoso, Minas Gerais State, Brazil. To our best knowledge, the majority of the studies using the Brazilian PEC-PCD criterion are conducted taking into consideration relatively small areas, that is, with site-specific conditions in terms of topography and land use and land cover. Therefore, more regional evaluation (e.g., at the scale of biome) of existing DEMs are needed in order to include a wider range of landscape conditions.

In this context, this study aims to analyze the altimetric conformity and accuracy of the global NASADEM_HGT and AW3D30 version 3.1 DEM with the altitude data from the Brazilian geodetic stations located in the Cerrado biome. These two DEMs were selected because they are freely available on a global scale with spatial resolution of 1 arc second, widely used by the scientific communities (except the NASADEM_HGT, launched recently).

## 2. Background

### 2.1. Brazilian Geodetic System (SGB)

The Brazilian Geodetic System (SGB) has a set of points with geographic coordinates (latitude and longitude) and altitude, calculated according to precision geodetic models [17]. The geodetic database can be understood as the set of information of the reference stations that constitute the SGB. Such stations are mostly materialized with concrete landmarks containing metal sheets on their top with identification of the respective registration and the type of station. The coordinates, altitude, and gravity of these stations are determined through the high-precision geodetic procedures and models [18].

The Decree-Law No. 243 of 28 February 1967 [19] established the guidelines and basis for the Brazilian cartography, recommending the development of a planialtimetric system of geodetic control points to serve as a basis for the development of cartographic works and to be the reference for the determination of coordinates and altitudes in Brazil. The Brazilian Institute of Geography and Statistics (IBGE) is responsible for the maintenance and densification of the SGB network [19]. The first geodetic basis was installed in 1944, near the city of Goiânia, Goiás State. In 1991, IBGE acquired Global Positioning System (GPS) receivers and began to use this technology to increase the SGB dataset, which consists of the following networks [20]:

1. Planialtimetric network: set of satellite-based geodetic stations, classified as GPS or Doppler, and the polygonal stations and triangulation vertices based on conventional surveying [20].
2. Altimetric network: set of reference levels for vertical positioning and composed of high precision geometric leveling measurements [21]. In 2018, this network was adjusted by geopotential numbers, where gravity observations in reference levels were considered with the objective of obtaining physically meaningful altitudes, resulting in normal–orthometric altitudes [22]. In this study, it was not possible to use the SGB’s altimetry network as a reference, determined through the reference levels, since IBGE has not launched the quasi-geoidal model yet to which the normal orthometric altitudes of the reference levels will be referred. This quasi-geoidal model is necessary for the conversion of the orthometric altitudes of the DEMs to the reference system of the IBGE´s planialtimetric stations used in the study.
3. Gravimetric network: a set of geodetic stations, called gravimetric stations, which contain information of the gravity acceleration and stations’ characteristics [23].

### 2.2. Brazilian Standard of Positional Accuracy

The Decree-Law No. 89817 of 1984 [24] details the Brazilian standard of positional accuracy for analog cartographic data (PEC) and, with adaptations to PEC-PCD (for digital cartographic products), constituted the Standard of Cartographic Accuracy (PEC) and Standard Error (EP) tolerances defined according to the eight scales of evaluation (1:1000, 1:2000, 1:5000, 1:10,000, 1:25,000, 1:50,000, 1:100,000, and 1:250,000) and the corresponding classes A (best accuracy), B, C, and D (worst accuracy). 

In 2010, the Brazilian Army Geographic Service (DSG) published the document titled Specifications of the Vector Geospatial Data Acquisition Techniques (ET-ADGV) [25]. In 2016, Technical Specification for Quality Control of Geospatial Data (ET-CQDG) [26] was published to provide a standardized way to evaluate the quality of the geospatial datasets that are part of the Brazilian National Cartographic System. Both the ET-ADGV and the ET-CQDG technical specifications are complementary to the Decree-Law No. 89817, which has regulatory power. In other words, a spatial data, to be classified in a certain scale and class, need to consider the following conditions [24,27]:

- Ninety percent of the samples points in a cartographic product shall present values of positional discrepancies equal to or less than the PEC tolerance value (1.6449*EP) of the scale and class tested, when compared with corresponding ground truth data.
- The RMSE of the positional discrepancies must be equal to or less than the EP tolerance defined for each scale and class.

Table 1 shows the error tolerances in meters of PEC-PCD, discriminated by class A, B, C, and D. It also shows the EP tolerances, which is related to the RMSE, of the positional discrepancies of the analyzed points in relation to the reference points [25].

For example, a specific DEM will be classified as class A in the 1:50,000 scale if it presents RMSE ≤ EP of 3.33 m and if 90% of altimetric discrepancies (errors) are ≤5.50 m (permissible error, represented in Table 1 by the PEC, in meters). Altimetric discrepancies (error—ε) is defined in this technical specification as the difference between DEMs’ altitudes (*Z_m_*) and the reference ones (*Z_r_*) (Equation (1)).

(1) $$\varepsilon =Z_{m}-Z_{r}$$

Carvalho and Silva [28] stated that both PEC and PEC-PCD do not clearly present the methodological procedures to be applied in the quality assessment process so that many complementary methods of analysis have been used to verify positional accuracies. Furthermore, the Decree-Law No. 89817 of 20 June 1984 defines that the PEC is a statistical dispersion indicator, relative to 90% of probability, corresponding to 1.6449 times the EP, that is, PEC = 1.6449*EP, considering this value relative to the 90% probability within the normal distribution curve. Thus, both PEC and PEC-PCD are valid only if the variable has a normal distribution.

## 3. Materials and Methods

### 3.1. Study Area

**Figure 1:**
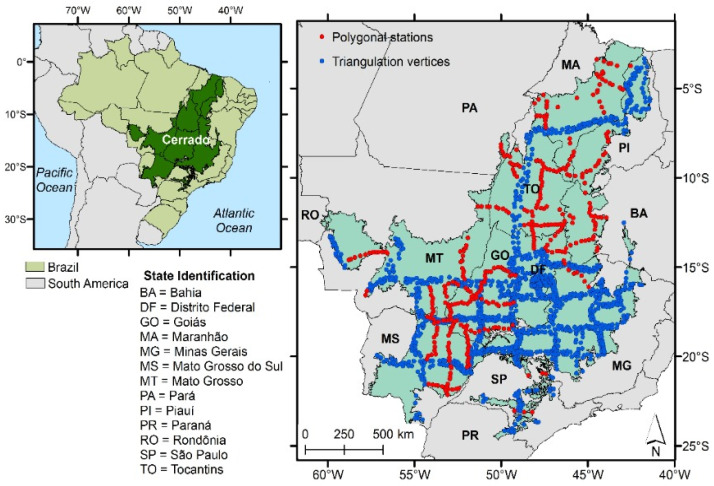
Location of the study area (Cerrado biome) in Brazil and the 1695 reference stations (Brazilian Institute of Geography and Statistics’ (IBGE’s) polygonal stations and triangulation vertices).

The Cerrado biome (Figure 1) occupies an area of 198.5 million hectares, i.e., about 23% of the Brazilian territory and covers, partially or totally, 1434 municipalities in 12 states, and the Federal District [29]. This biome corresponds to the world’s richest tropical savanna in terms of biodiversity, is considered as one of the world’s hotspots for biodiversity conservation [30], and encompasses eight headwaters of the twelve most important hydrographic basins in Brazil [31]. Besides, the Cerrado plays an important role in the Brazilian agriculture, with the annual crops cultivated in this biome representing 40% of the national production [31]. In the last four decades, the annual crops that expanded mostly in the Cerrado were soybean, corn, cotton, sugarcane, sorghum, and rice. Other crops such as coffee, tomatoes, beans, garlic, peanuts, and potatoes also have expanded, reinforcing the biome’s prominent agricultural position in the country [32].

Agricultural modernization, intensified after the 1970s, marked the beginning of the fast process of Cerrado’s native vegetation conversion into areas for production of agricultural commodities, which caused approximately 50% loss in its native vegetation [33]. Thus, it is necessary to understand its dynamics to protect its natural resources and to guarantee environmentally sustainable exploitation of this ecosystem.

### 3.2. Planialtimetric Reference Data

Data from the polygonal stations and triangulation vertices, which compose the planialtimetric geodetic network of the SGB, were used as reference. We found 387 polygonal stations and 1308 triangulation vertices with altitude data, resulting in 1695 stations with orthometric altitude data referenced to the Imbituba vertical datum and SIRGAS 2000 horizontal datum, over the Cerrado biome (Figure 1).

### 3.3. Digital Elevation Models

#### 3.3.1. NASADEM_HGT

NASADEM expanded the legacy of the SRTM by improving the accuracy of the DEM and data coverage on a global scale. SRTM mission mapped the topography of continental areas of the Earth between 60° N latitude and 56° S latitude using InSAR [3,34,35]. Three versions of the SRTM were launched; the last one is the SRTM Global 1 arc second, version 3.0 (SRTM Plus or SRTM NASA Version 3), which is void-filled using elevation data from ASTER GDEM 2, USGS National Elevation Dataset, and USGS Global Multi-resolution Terrain Elevation Data (GMTED) 2010 [36,37]. SRTM Plus was produced under the NASA’s Making Earth System Data Records for Use in Research Environments (MEaSUREs) Program and, in 2014, it was publicly announced with a resolution of 1 arc second (~ 30 m in the equator line) [37]. The performance requirements for the worldwide SRTM data products need to reach vertical absolute height errors below 16 m for 90% of the data (LE90) (RMSE of 9.73 m) [38,39,40,41] and circular absolute geolocation errors below 20 m for 90% of the data (CE90) [40]. The LE90 for South America was reported by [41] as 6.2 m, which corresponds to an RMSE of 3.8 m. 

Improvements have been made by reprocessing original SRTM raw signals data using enhanced algorithms and by incorporating data derived primarily from the Ice, Cloud, and Land Elevation Satellite (ICESat) Geoscience Laser Altimeter System (GLAS) and ASTER GDEMs. The LiDAR GLAS instruments of the ICESat mission and ASTER DEMs were not available during the original SRTM processing [42,43]. The Land Processes Distributed Active Archive Center (LP DAAC) is responsible for the archiving and distribution of NASA’s MEaSUREs program. Five NASADEM products are made available to the academic and scientific community, differing by type (Table 2).

In this study, we used the global NASADEM Merged DEM version 1 product (NASADEM_HGT), distributed in 1° by 1° tiles with 1 arc second spatial resolution (~30 m in Equator line), and referenced to the EGM96 geoid model. The layers included the DEM itself, the number of scenes (NUM), and the SRTM-based water body mask (SWB). The NUM layer indicates the number of DEM tiles that were processed for each pixel and the source of the data [43]. As the NASADEM_HGT model is a new release and was made available to the public recently (April 2020), there is little information in the literature specifically for this DEM regarding its validation, for vertical and horizontal absolute and relative errors.

#### 3.3.2. AW3D30

The DEM called AW3D30 was launched by the Japan Aerospace Exploration Agency (JAXA) with a spatial resolution of approximately 30 m in the equator line, resulting from the resampling of the ALOS World 3D DEM (AW3D) data, with 0.15 arc second (~ 5-meter spatial resolution). In five years of operation, ALOS produced approximately 6.5 million scenes covering the entire globe, with horizontal and vertical accuracy—5 m (RMSE)—being reported only for the 5-m dataset [2,44] (Table 3). This DEM has orthometric altitudes referenced to the EGM96 geoid model [45]. In this study, we used the version 3.1, launched in April 2020. It has different pixel spacing for each latitude zone at high latitudes, improved coastline and a new additional data for filling voids [46].

### 3.4. Validation

While the NASADEM_HGT product has 3601 × 3601 pixels per 1° × 1° grid with overlapping in the borders, the AW3D30 has 3600 × 3600 pixels per 1° × 1° grid without overlapping in the borders, resulting in a displacement of 1/2 pixel. Thus, it was necessary to perform displacement to make the pixels coincident, which was done through a Python language script using GDAL/OGR library [47].

In order to evaluate the DEMs, it was necessary to make them comparable to the IBGE reference stations. Thus, the orthometric altitudes referenced to the EGM96 geoid model was converted to the orthometric altitudes referenced to the Imbituba vertical datum and SIRGAS 2000 horizontal datum. The following steps were performed, based on the methodologies employed by [2,48,49].

**Figure 2:**
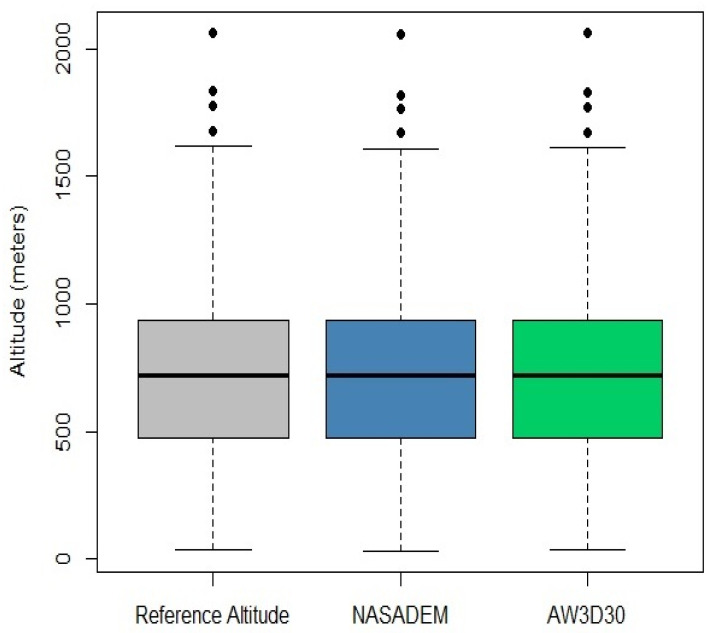
Boxplots of reference altitude, the NASADEM Merged Digital Elevation Model Global 1 arc second (NASADEM_HGT), and the Advanced Land Observing Satellite Global Digital Surface Model (ALOS AW3D30) datasets from the Cerrado biome.

- Computation of the geoidal undulation of the EGM96 model from a 15′ grid file provided by the National Geospatial Intelligence Agency (NGA). A 30-meter spatial resolution grid was generated using the Spline interpolation method available in the GRASS software [50].
- Calculation of the ellipsoidal height (*h*), obtained by the sum of the geoid undulation (*N*) and the orthometric height *H* (*h* = *H* + *N*) [51]. *H* (datum: EGM96) was converted into *h* (datum: WGS84) based on the addition of the EGM96 geoid undulation values obtained in the previous step.
- Computation of the geoidal undulation for the determination of orthometric altitude (Imbituba vertical datum) using input grid of the MAPGEO2015 software (5′ interval). This file was also interpolated to 30 m using the Spline interpolation method in the GRASS software.
- Conversion of the ellipsoidal altitude (WGS84) into the orthometric altitude referenced to the Imbituba vertical datum based on the subtraction of the geoidal undulation values obtained in the previous step.
- Conversion of the WGS84 horizontal datum to SIRGAS 2000 horizontal datum.
- The same script was also used to obtain the altitudes of the resulting raster GRID over the reference stations. These values were described in terms of mean, standard deviation, quartiles, and coefficient of variation, as well as with the support of boxplots, scatterplots, and histograms. We detected the presence of four outliers in the DEM models and in the reference altitudes (Figure 2), indicating equivalence among these outliers and corroborating the impression of high conformity among these datasets, i.e., the distribution of these three datasets is practically the same.

The normality of the dataset was verified using the Shapiro–Wilk and Anderson–Darling tests [52,53]. The data did not follow normal probability distribution, with p-values 0.05 (Table 4). Pearson’s correlation coefficients (*r*) were also calculated to check the level of correlation among the data obtained by the two DEMs and the reference stations. Positional errors were assessed according to the parameters recommended in the PEC-PCD regulated by the Decree-Law No. 89817 of 20 June 1984, as well as in the technical specification for the acquisition of vector geospatial data (ET-ADGV) and in the technical specification for quality control of geospatial datasets products (ET-CQDG), complementary to the Decree-Law (Table 1).

The GeoPEC software [54] was used to classify DEMs according to accuracy measurements such as mean square error (*MSE*), proposed by Gauss and known as (Equation (2)).

(2) $$MSE=E\;\left[{\left(\hat{\theta }-\theta \right)}^{2}\right]=\sigma_{\hat{\theta }}^{2}+b^{2}\;≅\frac{\sum_{i=1\;}^{N}\varepsilon_{i}^{2}}{N}+b^{2}$$

where *σ*^2^ represents the dispersion of the measurements (variance or uncertainty) and *b* represents the tendency, error of estimator, or bias. In other words, *σ*^2^ and *b* represent random and systematic errors, respectively. For large samples, the *MSE* corresponds to the quadratic mean of the errors (*ε*) [55,56].

Although this is a proposed measure of accuracy, a better way to evaluate it is in terms of the independent parameters of tendency and precision (*σ*), which allows discrimination between systematic and random errors. Since there is no tendency, accuracy and *σ* are confused. Mathematically, the tendency is the mean of the altimetric discrepancies—difference between the observations (measured/estimated) and the known (or expected reference) values [56,57]—and the *σ* is the standard deviation of errors. In this study, *b* and *σ* were calculated by Equation (3) and Equation (4), respectively. The accuracy was calculated by the *RMSE* (Equation (5)).

(3) $$b=\frac{\sum_{i=1}^{N}\varepsilon_{i}}{N}$$

(4) $$\sigma =\sqrt{\frac{\sum_{i=1}^{N}{\left(\varepsilon_{i}-b\right)}^{2}}{N-1}}$$

(5) $$RMSE=\sqrt{\frac{\sum_{i=1}^{N}\varepsilon_{i}^{2}}{N}}$$

In addition to these statistical measurements, Pearson’s correlation coefficient (*r*) was employed to verify the correlation between DEMs’ altitudes and reference data and the Willmott index of agreement (*d*) [58] was obtained.

### 3.5. Comparison of DEMs in Eight Municipalities with the Highest Grain Production in the Cerrado

**Figure 3:**
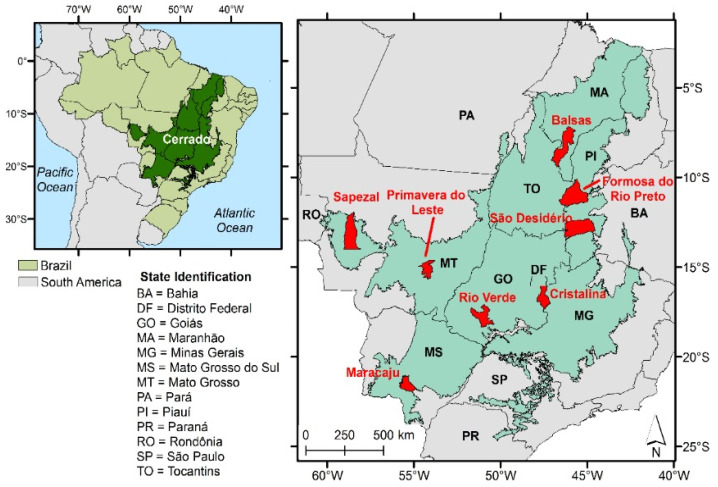
Eight selected municipalities in the Cerrado biome with the highest grain production.

**Figure 4:**
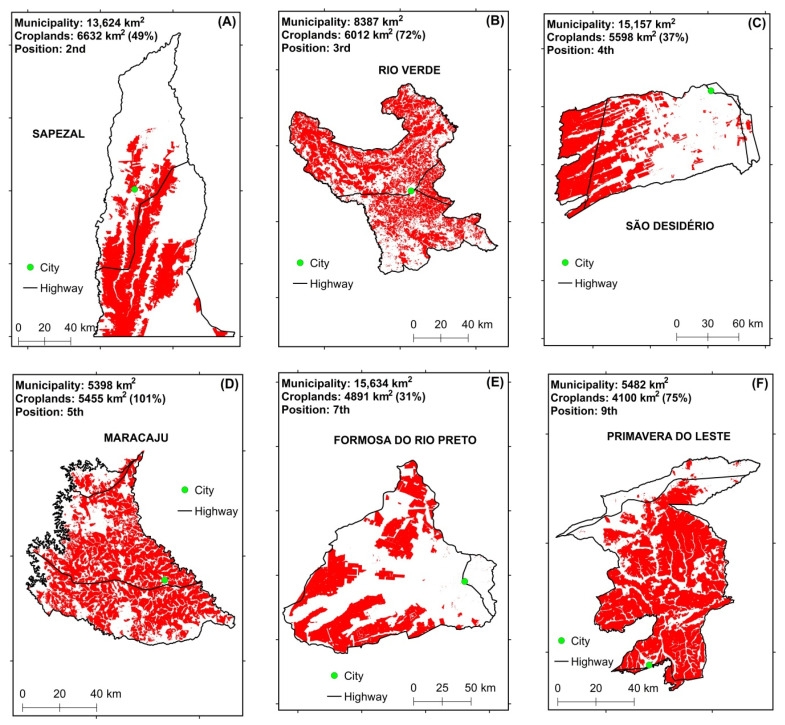

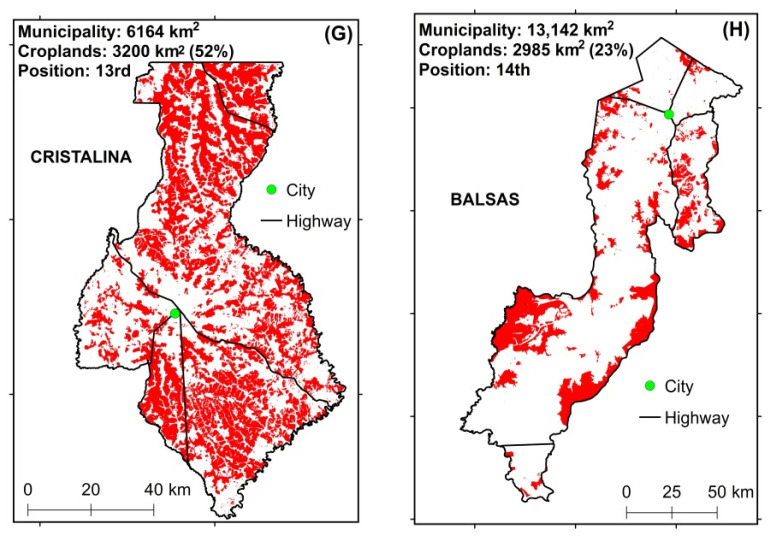
Areas with annual and perennial crops in 2018 (in red) in the municipalities of Sapezal, Mato Grosso State (**A**), Rio Verde, Goiás State (**B**), São Desidério, Bahia State (**C**), Maracaju, Mato Grosso do Sul State (**D**), Formosa do Rio Preto, Bahia State (**E**), Primavera do Leste, Mato Grosso State (**F**), Cristalina, Goiás State (**G**), and Balsas, Maranhão State (**H**). Source: [59]. In the Maracaju municipality, the area of croplands is larger than the area of municipality probably because of the double cropping system.

Eight municipalities with the highest agricultural production in the Cerrado biome [59] were selected to compare the two DEMs original values with each other (Figure 3; Table 5). The spatial distribution of these municipalities in the biome and their altitudes were also taken into account to guarantee spatial representativeness of them in the biome. The temporary and perennial crops mapped in 2018 by the MapBiomas Project [60] were used as a mask. The altitudes were analyzed pixel by pixel within these areas (Figure 4). The MapBiomas Project generates historical series of annual land use and land cover maps from Brazil based on cloud processing and automated classifiers in the Google Earth Engine platform.

Initially, a script using Python language and GDAL/OGR library was written in order to obtain the pixel values of the analyzed models [47]. Descriptive measurements of the models were obtained for agricultural areas of each municipality in terms of boxplots, histograms, and scatterplots. In addition, the corresponding *r* values were also calculated to quantify the level of correlation between the models. After the descriptive analysis of the data, the altitude measurements obtained through the models were compared with each other. This comparison was made by calculating the differences between the average altitudes through Cohen’s effect size (*dc*) [61]. 

## 4. Results

### 4.1. Descriptive Analysis of DEMs Against the Reference Stations

**Figure 5:**
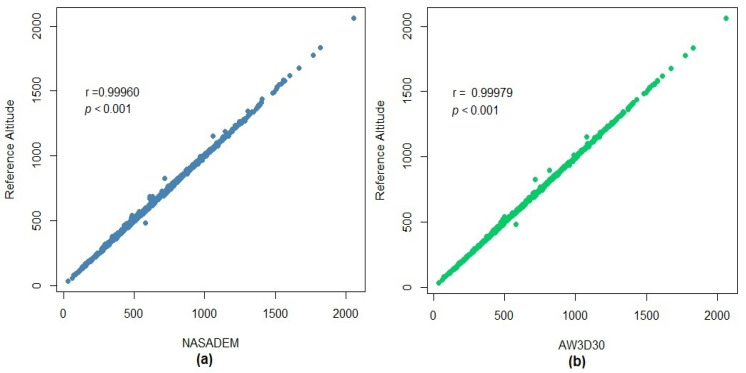
Relation between reference altitudes and those estimated by the NASADEM_HGT (**a**) and AW3D30 (**b**) models.

Descriptive statistical measures of the reference altitudes and DEMs are shown in Table 6. The altitudes obtained for the NASADEM_HGT and AW3D30 were quite close with the measures provided by the SGB. In Figure 5, it is also possible to note a strong linear relationship between the reference altitudes and the estimations from NASADEM_HGT and AW3D30, which is emphasized by the *r* values close to 1: 0.99960 and 0.99979 for NASADEM_HGT and AW3D30, respectively. The Willmott index of agreement (*d*) was also very close to 1: 0.99978 for the NASADEM; and 0.99989 for the ALOS AW3D30.

**Figure 6:**
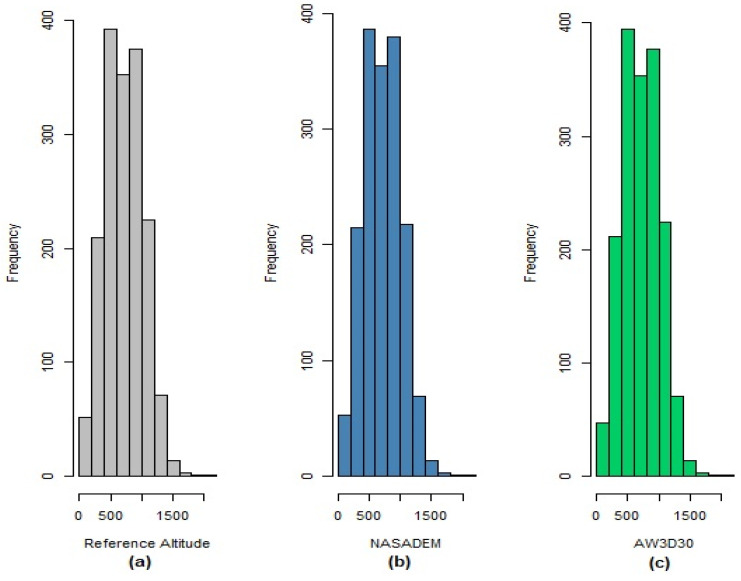
Histograms of altitudes from reference (**a**), from NASADEM_HGT (**b**), and AW3D30 (**c**) digital elevation models.

Figure 6 shows the histograms of the reference altitudes and the NASADEM_HGT and AW3D30 models. It is possible to infer that the distributions of the altitudes measured by the NASADEM_HGT and AW3D30 are practically identical to the distribution of the reference altitudes, indicating high conformity of the models.

### 4.2. Classification of DEMs Considering PEC-PCD

The evaluation of the PEC-PCD was conducted in two steps. First, the RMSE was calculated for each DEM in order to compare them to the EP as a function of the scale. The RMSEs of the models were 8.87783 m and 6.14927 m for NASADEM_HGT and AW3D30, respectively. The EP for the 1:100,000 scale and class A is 8.33 m and for the 1:100,000 scale and class B is 16.66 m (Table 7). According to this criterion, the NASADEM_HGT meets the 1:250,000 scale and class A, and 1:100,000 scale and class B. The AW3D30 meets the 1:100,000 scale and class A, and 1:50,000 scale and class B. At least 90% of the points presented altitude errors equal or lower than the PEC values shown in this table. In this step, the two DEMs met the 1:100,000 scale and class A. However, when analyzing both criteria together, considering the class A, it was possible to conclude that the NASADEM_HGT meets 1:250,000 scales while AW3D30 meets the 1:100,000 and lower scales, considering the class B, NASADEM_HGT meets the 1:100,000 scale while AW3D30 meets 1:50,000 and lower scales.

The positional accuracy of the two models was also verified through the GeoPEC software which analyzes whether the discrepancies are equal or lower than the PEC and if RMSE ≤ EP, according to the assumptions of Decree-Law No. 89817 of 20 June 1984 and the specifications of ET-CQDG. The same results and classifications described in the previous paragraphs were obtained. The normality of altitude discrepancies was verified using the Shapiro–Wilk and Anderson–Darling tests. The data did not follow a normal distribution, according to the *p*-values (0.05) (Table 8).

Table 9 presents the results of the statistical analyzes in relation to the altitude discrepancies. Overall, AW3D30 presented better results, though with very close values regarding the minimum and maximum errors, while the maximum errors were the same in both models.

**Figure 7:**
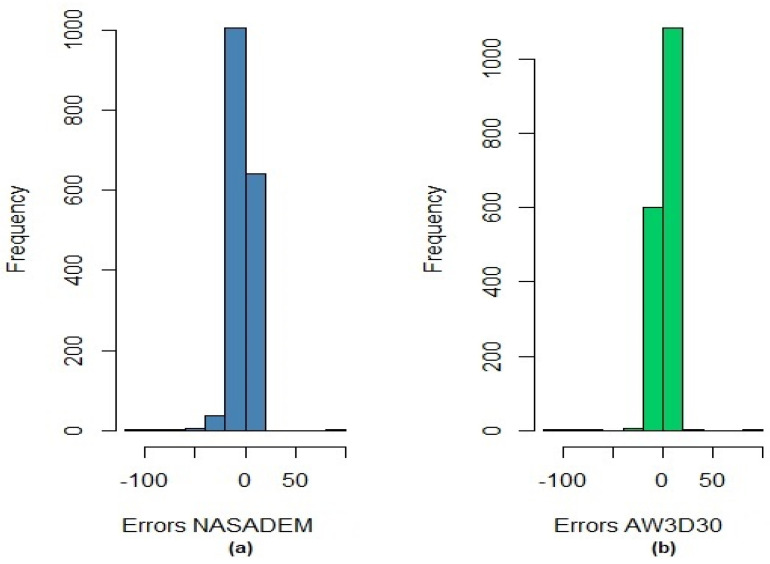
Histograms of altitude discrepancies for NASADEM_HGT (**a**) and AW3D30 (**b**).

The mean error or bias, representing the tendency, was −2.90 m for NASADEM_HGT and 0.69 m for AW3D30, indicating that the AW3D30 data presents smaller systematic error than the NASADEM_HGT data, and the altitude values represented in the NASADEM_HGT model are underestimated in relation to the reference and overestimated in relation to the AW3D30. The result for AW3D30 is close to the ideal (zero), and for NASADEM_HGT is low considering the spatial resolution and the appropriate scales for the models. The standard deviation (precision) was lower for the AW3D30, indicating that the discrepancies for this model are closer to the average of the discrepancies. The precision of this model (6.11 m) is 1.37 times greater than the NASADEM_HGT precision (8.39 m), which means that altitude errors are less scattered in the AW3D30 than in the NASADEM_HGT. The histograms referring to the errors for the altimetric component of the DEMs are presented in Figure 7.

The RMSE for the AW3D30 was 6.15 m and for the NASADEM_HGT was 8.88 m. In relation to the RMSE, understanding accuracy as a reflection of the tendency and precision measures and, as the tendency value in AW3D30 was very close to 0 and low in NASADEM_HGT, the accuracy value (in this study represented by the RMSE) tends to be similar to the precision values (represented by standard deviation in this study).

### 4.3. Descriptive Analysis of DEMs in the Municipalities with the Highest Grain Production in the Cerrado

**Figure 8:**
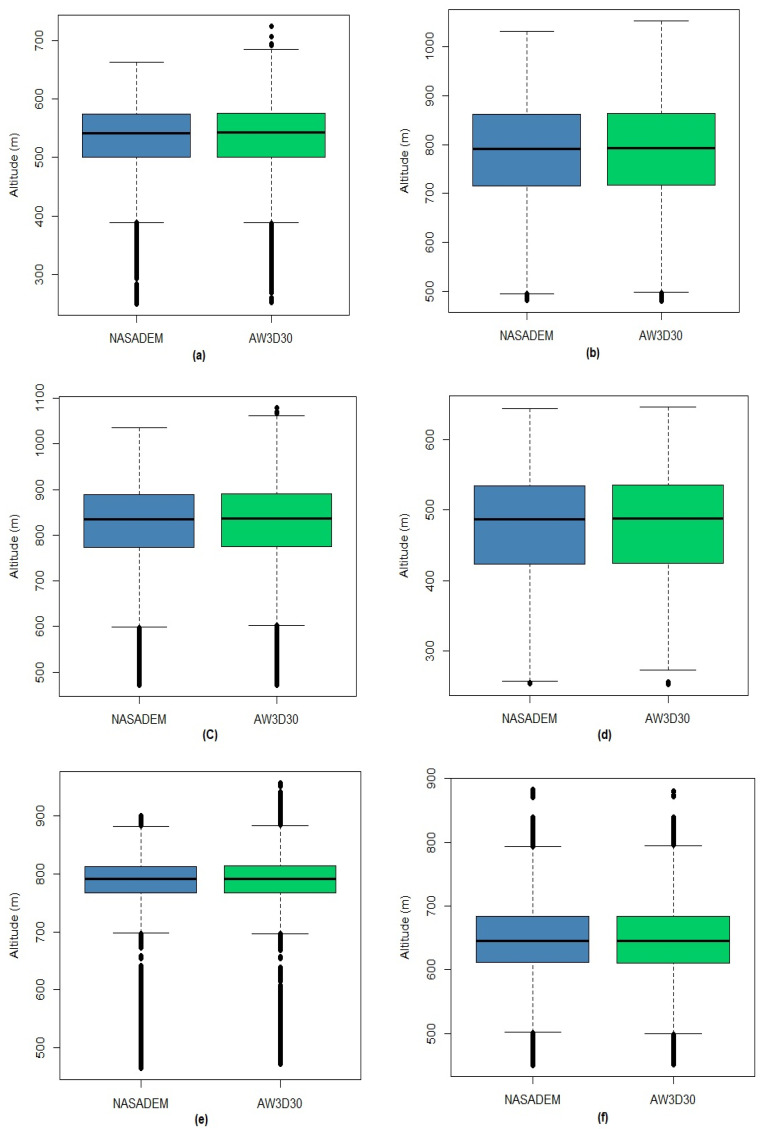

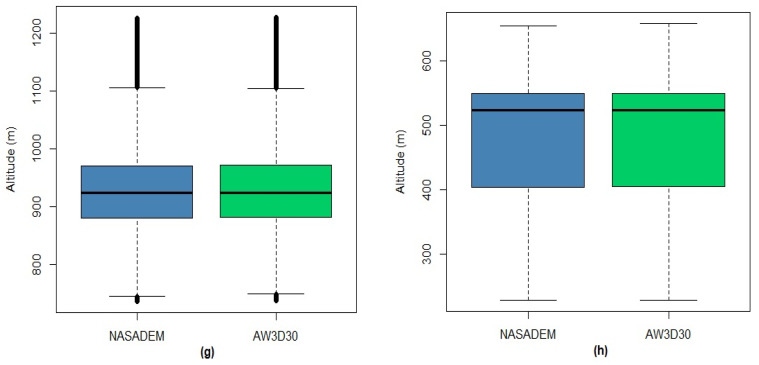
Boxplots of the NASADEM_HGT and AW3D30 digital elevation models for the agricultural areas of Sapezal (**a**), Rio Verde (**b**), São Desidério (**c**), Maracaju (**d**), Formosa do Rio Preto (**e**), Primavera do Leste (**f**), Cristalina (**g**), and Balsas (**h**) municipalities.

Table 10 presents the descriptive measures for each municipality with the highest harvested area in the Cerrado biome. Considering the values of means, standard deviations, quartiles, and coefficients of variation, the values are very close between the NASADEM_HGT and AW3D30 models; so, the initial impression is that the DEMs have very similar altitudes, with homogenous data according to the coefficient of variation (CV (%)). Figure 8 shows the boxplots for agricultural areas in each municipality, with NASADEM_HGT represented in blue and AW3D30 in green. Analyzing them, it is possible to realize that the distributions of the measurements of each model are very similar, a fact that is common for each municipality analyzed.

**Figure 9:**
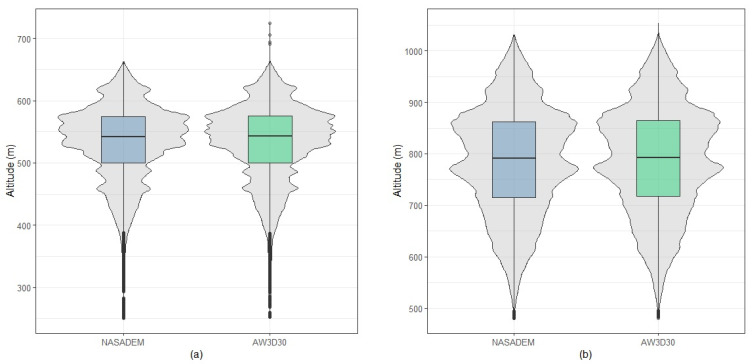

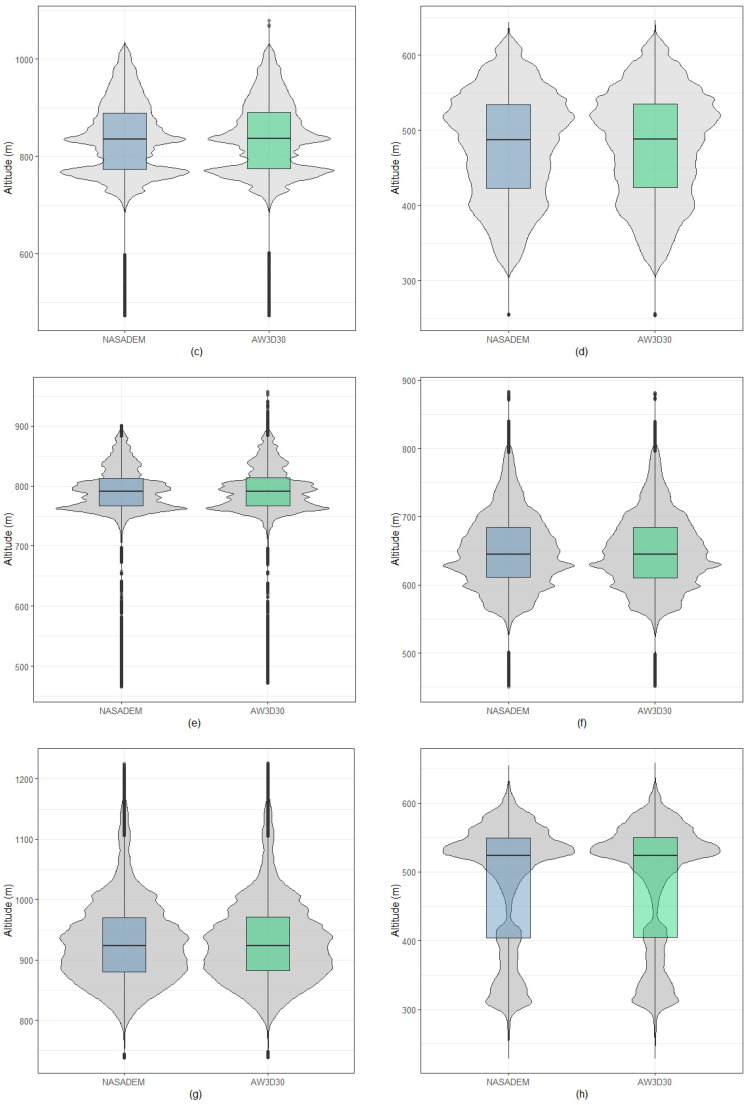
Violin plots of the NASADEM_HGT and AW3D30 digital elevation models for the agricultural areas of Sapezal (**a**), Rio Verde (**b**), São Desidério (**c**), Maracaju (**d**), Formosa do Rio Preto (**e**), Primavera do Leste (**f**), Cristalina (**g**), and Balsas (**h**) municipalities.

Figure 9 shows the violin plots, which combine boxplots and histograms in the same graphical output of chosen municipalities. It appears that the distributions of measurements inherent to the NASADEM_HGT and AW3D30 are very similar. Such graphs confirm the initial impression that the models tend to present values of very close altitudes. Overall shape and distribution are similar for both models, with quartiles very close to each other, where it is possible to observe the correspondence of outliers. 

**Figure 10:**
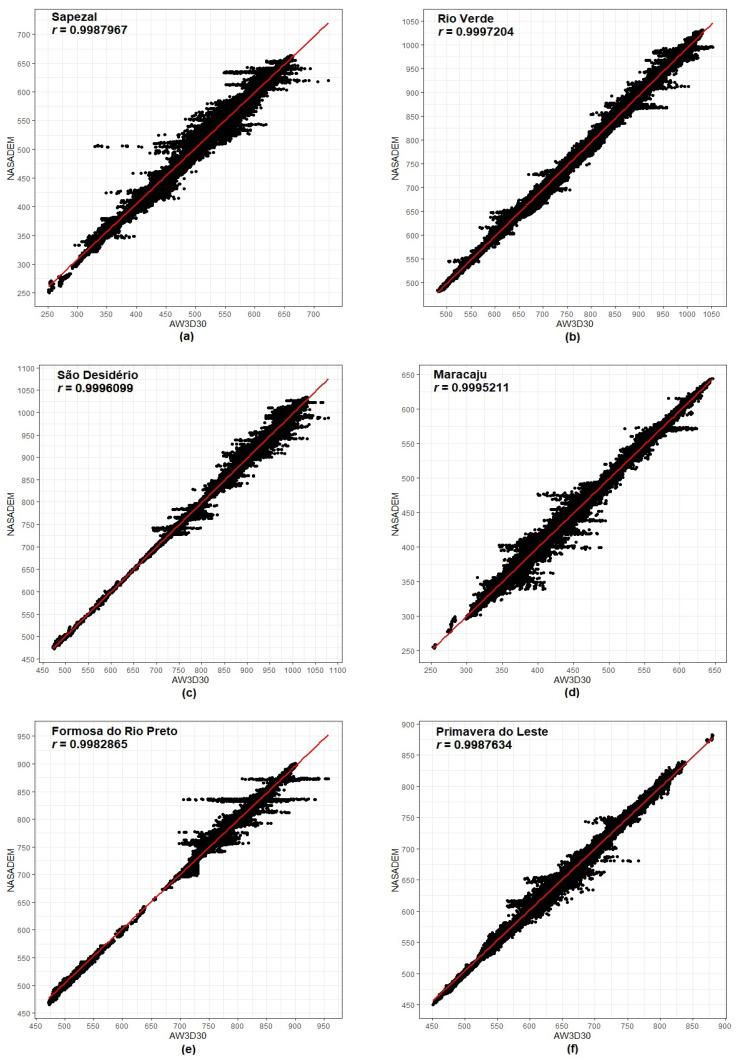

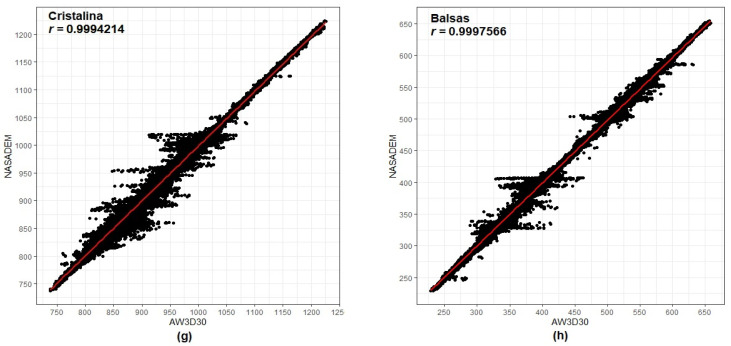
Relation between NASADEM_HGT and AW3D30 for the agricultural areas of Sapezal (**a**), Rio Verde (**b**), São Desidério (**c**), Maracaju (**d**), Formosa do Rio Preto (**e**), Primavera do Leste (**f**), Cristalina (**g**), and Balsas (**h**) municipalities.

Figure 10 shows the scatterplots for each municipality in order to assess the degree of association between the altitudes of each model. It is possible to note that there is a linear relation between the variables analyzed and values very close to 1 (*r* 0.99), indicating a correlation quasi-perfect between the altitudes of the two models for all municipalities analyzed.

### 4.4. Comparison of DEMs in Agricultural Areas of Municipalities with the Highest Grain Production in the Cerrado Using the Size of Cohen Effect (dc)

In this subsection, the results of the comparison between the altitudes of the NASADEM_HGT and AW3D30 models, performed by measuring the size of the Cohen effect (*dc*), are presented. The interpretations of the results were performed according to Table 11, while the measurements of size effects for each municipality are shown in Table 12. It is possible to notice that all values for the size of the Cohen effect (*dc*) were less than 0.19. Thus, it can be said that the effect of the difference between the altitude measurements of the NASADEM_HGT and AW3D30 models is insignificant for all the analyzed municipalities. These results, combined with the descriptive analysis, allow us to conclude that the two DEMs tend to provide very similar altitude measurements.

## 5. Discussion

The adopted methodology allowed us to conclude that the vertical accuracy of the AW3D30 (RMSE of 6.15 m) is close to that specified for the original ALOS World 3D (~5 m of spatial resolution) (RMSE of 5 m). The vertical accuracy of the NASADEM_HGT (RMSE of 8.88 m) is in accordance with the vertical accuracy requirements for the overall product of SRTM mission, from where its data is derived, 16 m (LE90), which corresponds to an RMSE of 9.76 m.

The tendency (bias) of the two models was −2.90 m and 0.69 m for NASADEM_HGT and AW3D30, respectively, which means that they have low and very low systematic underestimation and overestimation errors, considering the nature of the biophysical variable under analysis (altitude) and the scales compatible with the models. The *r* and *d* values between the altitudes of the DEMs and the reference altitudes were very close to 1, that is, an almost perfect correlation and agreement.

For the Cerrado biome, more than 90% of the altitude discrepancies between IBGE data and the NASADEM_HGT and the AW3D30 models were less than 13.7 m. However, the AW3D30 presented lower RMSE, fitting in the class B of 1:50,000 scale and in the class A of 1:100,000 scale, and smaller. The NASADEM_HGT is aligned with the class B of the 1:100,000 scale and, for class A, it meets the 1:250,000 scale. A similar classification was found by [63] when AW3D30 DEM was analyzed considering the PEC-PCD in different regions of Brazil.

It is important to take into account the scales of each model pointed out through the classification based on the PEC-PCD carried out in this study and the non-use of the DEMs in scales larger than those pointed out, as they are not compatible. The AW3D30 presented lower values of RMSE (higher accuracy), standard deviation (higher precision), and bias. Therefore, it is more accurate and precise than the NASADEM_HGT considering the methodology and dataset adopted in this study. Previous authors comparing datasets from SRTM and AW3D30 with reference points surveyed in the field [49,63] and with more accurate DEMs [2] also found similar results in relation to accuracy. Uuemaa et al. [11] compared vertical accuracy of freely available global DEMs: ASTER, AW3D30, MERIT, TanDEM-X, SRTM, and NASADEM in Estonia, Norway, New Zealand, and China and concluded that the AW3D30 was the most robust and presented the most stable performance in most of the tests employed. It is, therefore, the best choice for a global analysis. According to these authors, the NASADEM model, as a successor of SRTM, showed only slight improvement in comparison to SRTM. These studies presented RMSE similar to those found in the present study for NASADEM and AW3D30.

When analyzing the DEMs with each other in different municipalities, it was possible to conclude that the two models present measurements of similar altitudes. The *r* values were very close to 1 for all eight municipalities analyzed and the effect of the difference in mean altitudes of the NASADEM_HGT and AW3D30 models, measured by the Cohen effect (*dc*), was insignificant, which corroborates that statement.

It is important to highlight the importance of the process of converting the elevations of the DEMs adopted in this study. Originally, the orthometric values referenced to the geoid model EGM96 and horizontal datum WGS 84 were transformed to ellipsoidal altitudes referenced to the ellipsoid WGS 84. Next, the transformation to orthometric altitudes referenced to the Imbituba vertical datum and SIRGAS 2000 horizontal datum, the same system of reference IBGE points, was done. This process allowed the comparison of the same type of measure in the same reference system.

As the data distributions used in this study did not show normal distribution, it would be interesting to consider, in the future, the classification of models according to standards that contemplate the situation of non-normal distribution for discrepancy in altimetric data such as the ASPRS standards [64]. It is also worth emphasizing the relevance of carrying out studies on regional scales such as the one adopted in this work, which brings a broader scope of analysis. This study is a precursor to NASADEM_HGT’s accuracy analysis for a regional scale, given its recent launch. The most recent version (3.1) of global DEM ALOS AW3D30 was also employed in this study.

## 6. Conclusions

In the Cerrado biome, the NASADEM_HGT and AW3D30 DEMs show statistically insignificant difference, according to the Cohen effect size and the correlation coefficient data. However, when analyzed by the PEC-PCD criterion, the AW3D30 data show better altimetric quality. NASADEM_HGT meets the 1:250,000 scale and class A (tolerance error of 16.67 m), and 1:100,000 scale and class B (tolerance error of 16.66 m). The AW3D30 meets the 1:100,000 scale and class A (tolerance error of 8.33 m), and 1:50,000 scale and class B (tolerance error of 6.66 m). Bias effects are found for both products, expressed by the vertical shift component. This effect is lower for the AW3D30 data than for the NASADEM_HGT data.

## Figures and Tables

**Table 1:** Standard of altimetric cartographic accuracy of digital elevation models (DEMs) for the production of digital cartographic products defined for eight different scales and for classes A (most restricted), B, C, and D (least restricted).

Scale	Class							
	A		B		C		D	
	PEC (m)	EP (m)	PEC (m)	EP (m)	PEC (m)	EP (m)	PEC (m)	EP (m)
1:1000	0.27	0.17	0.50	0.33	0.60	0.40	0.75	0.50
1:2000	0.27	0.17	0.50	0.33	0.60	0.40	0.75	0.50
1:5000	0.54	0.34	1.00	0.66	1.20	0.80	1.50	1.00
1:10,000	1.35	0.84	2.50	1.67	3.00	2.00	3.75	2.50
1:25,000	2.70	1.67	5.00	3.33	6.00	4.00	7.50	5.00
1:50,000	5.50	3.33	10.00	6.66	12.00	8.00	15.00	10.00
1:100,000	13.70	8.33	25.00	16.66	30.00	20.00	37.50	25.00
1:250,000	27.00	16.67	50.00	33.33	60.00	40.00	75.00	50.00

Source: [25].

**Table 2:** NASADEM products and their groupings.

Product	Description	Data Type	Units	Fill Value	No Data Value	Valid Range	Scale Factor
**NASADEM_HGT: NASADEM Merged DEM Product Grouping**							
hgt	Void-filled DEM merge	2-byte signed integer	meters (relative to the EGM96 geoid)	N/A	N/A	−32,767 to 32,767	N/A
num	NUM file associated with hgt file	byte	Class: 1–255 (see “Reference Data for Number of Scenes Layer”)	N/A	N/A	0 to 255	N/A
swb	Updated SRTM water body data	byte	Class: 0 for land; 255 for water	N/A	N/A	255	N/A
**NASADEM_SC: NASADEM Slope and Curvature Product Grouping**							
slope	Slope derived from hgt	2-byte unsigned integer	hundreds of degrees (0 = water)	0	NaN	Non-negative	See Units
aspect	Slope aspect angle derived from hgt	2-byte unsigned integer	Hundreds of degrees clockwise from North (0 = water)	0	NaN	Non-negative	See Units
plan (planc)	Plan curvature derived from hgt	4-byte real	Inverse meters (0 = water)	0	NaN	-	N/A
profile (profc)	Profile curvature derived from hgt	4-byte real	Inverse meters (0 = water)	0	NaN	-	N/A
swbd (swb)	Updated SRTM water body data	byte	Class: 0 for land; 255 for water	N/A	N/A	255	N/A
**NASADEM_SSP: NASADEM SRTM Subswath Product Grouping**							
tot.cor	Radar total correlation	2-byte unsigned integer	correlation value × 10,000 (0 = void)	0	N/A	Non-negative	See Units
vol.cor	Radar volumetric correlation	2-byte unsigned integer	correlation value × 10,000 (0 = void)	0	N/A	Non-negative	See Units
img	Radar individual images	Byte	DN + 128 (0 = void) (i.e., fileValue = 10 × log10(actualValue) + 128)	0	N/A	-	See Units
Inc0	Radar incidence angle (relative to ellipsoid)	2-byte unsigned integer	hundreds of degrees (0 = void)	0	N/A	Non-negative	See Units
inc	Radar incidence angle (local)	2-byte unsigned integer	hundreds of degrees (0 = void)	0	N/A	Non-negative	See Units
**NASADEM_SIM: NASADEM SRTM Image Mosaic Product Grouping**							
Img_comb (img)	Radar combined images	byte	DN + 128 (0 = void) (i.e., fileValue = 10 x log10 (actualValue) + 128)	0	N/A	-	See Units
img_comb_num (img.num)	NUM file associated with combined images	byte	Number of pixels averaged for each img_comb output pixel	0	N/A	0 to 10	N/A
**NASADEM_SHHP: NASADEM SRTM-only Height and Height Precision Product Grouping**							
Hgt_srtmOnly (hgts)	SRTM-only floating-point DEM	4-byte real	meters (relative to the WGS84 ellipsoid)	−32,768	N/A	-	N/A
err	Height error (precision)	2-byte unsigned integer	millimeters (32,769 = void)	32,769	N/A	Non-negative	N/A

Source: [42]. N/A and NaN = No value.

**Table 3:** Characteristics and global accuracy (90% of the data (CE90), 90% confidence of vertical accuracy (LE90), and root mean square error (RMSE)) of the datasets used in this study.

Dataset	Imaging System	Wavelength	Pixel Spacing	Horizontal Accuracy	Vertical Accuracy
NASADEM_HGT	SAR C-band	5.66 cm	30 m	20 m (CE90)	16 m (LE90)
ALOS AW3D	PRISM	0.52–0.77 μm	5 m	5 m (RMSE)	5 m (RMSE)

Source: [2].

**Table 4:** Normality tests of reference altitudes and those obtained by the NASADEM_HGT and AW3D30 models.

Parameter	Test of Normality	
	Shapiro–Wilk	Anderson–Darling
Reference altitude	0.001	0.001
NASADEM_HGT	0.001	0.001
AW3D30	0.001	0.001

**Table 5:** Agricultural position and harvest area of soybean, corn, cotton, and sugarcane of chosen municipalities.

Municipality (State)	Position in the Cerrado in Terms of Grain Production	Harvested Area (ha)
Sapezal (MT)	2nd	663,198
Rio Verde (GO)	3rd	601,210
São Desidério (BA)	4th	559,763
Maracaju (MS)	5th	545,458
Formosa do Rio Preto (BA)	7th	489,137
Primavera do Leste (MT)	9th	410,000
Cristalina (GO)	13th	320,000
Balsas (MA)	14th	298,495

Source: [59].

**Table 6:** Quantitative measurements of reference altitude (m) and those obtained by the NASADEM_HGT and AW3D30 models. Total number of samples: 1695. Min = minimum altitude; Max = maximum altitude; SD = standard deviation. Q1 = first quartile; Q2 = second quartile; Q3 = third quartile; and CV (%) = coefficient of variation in percentage.

Variable	Min	Max	Mean	SD	Q1	Q2	Q3	CV (%)
Reference altitude	35.37	2061.76	716.48	297.78	476.66	721.81	936.28	41.56
NASADEM_HGT	31.77	2056.24	713.58	297.44	471.36	718.32	933.38	41.68
AW3D30	36.77	2061.24	717.17	297.37	474.51	719.04	936.16	41.46

**Table 7:** Percentage of points that presented discrepancies lower than the Cartographic Accuracy Standard (PEC) (m).

Scale	Class	Standard Error EP (m)	PEC (m)	Percentage (%)	
				NASADEM_HGT	AW3D30
1:25,000	A	1.67	2.70	49	60
1:25,000	B	3.33	5.00	69	85
1:50,000	A	3.33	5.50	72	87
1:50,000	B	6.66	10.00	86	96
1:100,000	A	8.33	13.70	92	99
1:100,000	B	16.66	25.00	98	100
1:250,000	A	16.67	27.00	99	100
1:250,000	B	33.33	50.00	100	100

**Table 8:** Tests of normality of the altitude discrepancies.

Variable	Test of Normality	
	Shapiro–Wilk	Anderson–Darling
NASADEM_HGT errors	0.001	0.001
AW3D30 errors	0.001	0.001

**Table 9:** Statistics of altitude discrepancies.

Parameters	NASADEM_HGT	AW3D30
Minimum error	−109.72	−108.72
Maximum error	96.80	97.80
Mean error or bias (tendency)	−2.90	0.69
Standard deviation (precision)	8.39	6.11
Root mean square error (RMSE) (accuracy)	8.88	6.15

**Table 10:** Quantitative measurements of altitude (m) extracted from NASADEM_HGT and AW3D30 over eight municipalities of the Cerrado with highest grain production. Min. = minimum altitude (m); Max. = maximum; SD = standard deviation; DEM = digital elevation model; Q1 = first quartile; Q2 = second quartile; Q3 = third quartile; and CV = coefficient of variation.

Municipality	DEM	Number of Pixels	Min. (m)	Max. (m)	Mean (m)	SD (m)	Q1 (m)	Q2 (m)	Q3 (m)	CV (%)
Sapezal	NASADEM	4,691,742	250	663	533.19	59.40	500	542	574	11.14
	AW3D30	4,691,742	252	724	533.60	59.86	500	543	575	11.22
Rio Verde	NASADEM	5,341,106	481	1031	786.07	103.38	715	791	862	13.15
	AW3D30	5,341,106	480	1053	788.03	103.56	717	792	864	13.14
São Desidério	NASADEM	6,222,247	472	1035	838.44	72.49	773	835	889	8.65
	AW3D30	6,222,247	472	1079	839.45	72.11	775	836	890	8.59
Maracaju	NASADEM	3,603,927	254	644	478.66	70.95	423	487	534	14.82
	AW3D30	3,603,927	253	646	479.71	71.14	424	488	535	14.83
Formosa do Rio Preto	NASADEM	5,696,443	465	901	794.13	35.74	767	791	813	4.50
	AW3D30	5,696,443	472	957	794.43	36.09	767	791	814	4.54
Primavera do Leste	NASADEM	3,511,471	450	883	649.37	53.43	611	645	684	8.23
	AW3D30	3,511,471	451	881	649.06	53.35	610	645	684	8.22
Cristalina	NASADEM	3,039,881	737	1225	930.38	69.26	880	923	970	7.44
	AW3D30	3,039,881	738	1226	931.26	68.99	882	924	971	7.41
Balsas	NASADEM	2,774,848	229	655	483.22	91.99	404	524	549	19.04
	AW3D30	2,774,848	229	658	484.29	91.71	405	524	550	18.94

**Table 11:** Interpretation of the Cohen effect size measurements.

Cohen Effect	Interpretation
≥1.30	Very high
0.80–1.29	High
0.50–0.79	Medium
0.20–0.49	Low
≤0.19	Not significant

Source: [62].

**Table 12:** Size measurements of the Cohen effect size per municipality.

Municipality	Mean Altitude		Mean Difference	Cohen Effect	Interpretation
	NASADEM_HGT	AW3D30			
Sapezal	533.19	533.60	0.40	0.00678507	Not significant
Rio Verde	786.07	788.03	1.95	0.01889108	Not significant
São Desidério	838.44	839.45	1.00	0.01388478	Not significant
Maracaju	478.66	479.71	1.05	0.01472942	Not significant
Formosa do Rio Preto	794.13	794.43	0.29	0.008158326	Not significant
Primavera do Leste	649.37	649.06	0.31	0.005793891	Not significant
Cristalina	930.38	931.26	0.89	0.01281434	Not significant
Balsas	483.22	484.29	1.07	0.01164616	Not significant

## Data Availability

Available upon request to the corresponding author.

## References

[1] Nikolakopoulos K.G. (2020). Accuracy assessment of ALOS AW3D30 DSM and comparison to ALOS PRISM DSM created with classical photogrammetric techniques. Eur. J. Remote Sens..

[2] Grohmann C.H. (2018). Evaluation of TanDEM-X DEMs on selected Brazilian sites: Comparison with SRTM, ASTER GDEM and ALOS AW3D30. Remote Sens. Environ..

[3] Farr T.G., Rosen P.A., Caro E., Crippen R., Duren R., Hensley S., Kobrick M., Paller M., Rodriguez E., Roth L. (2007). The Shuttle Radar Topography Mission. Rev. Geophys..

[4] Abrams M., Crippen R., Fujisada H. (2020). ASTER global digital elevation model (GDEM) and ASTER global water body dataset (ASTWBD). Remote Sens..

[5] Florinsky I.V., Skrypitsyna T.N., Luschikova O.S. (2018). Comparative accuracy of the AW3D30 DSM, ASTER GDEM, and SRTM1 DEM: A case study on the Zaoksky testing ground, Central European Russia. Remote Sens. Lett..

[6] Rizzoli P., Martone M., Gonzales C., Wecklich C., Tridon D.B., Brautigam B., Backmann M., Schulze D., Fritz T., Huber M. (2017). Generation and performance assessment of the global TanDEM-X digital elevation model. ISPRS J. Photogramm. Remote Sens..

[7] Crippen R., Buckley S., Agram P., Belz E., Gurrola E., Hensley S., Kobrick M., Lavalle M., Martin J., Neumann M. (2016). NASADEM global elevation model: Methods and progress. Int. Arch. Photogramm. Remote Sens. Spat. Inf. Sci..

[8] Tadono T., Nagai H., Ishida H., Oda F., Naito S., Minakawa K., Iwamoto H. (2016). Generation of the 30-m-mesh global digital surface model by ALOS PRISM. Int. Arch. Photogramm. Remote Sens. Spat. Inf. Sci..

[9] Buckley S. (2019). NASADEM_HGT v001 (NASADEM Merged DEM Global 1 Arc Second), EarthData, NASA. https://lpdaac.usgs.gov/products/nasadem_hgtv001/.

[10] Yap L., Kandé L.H., Nouayou R., Kamguia J., Ngouh N.A., Makuate M.B. (2018). Vertical accuracy evaluation of freely available latest high-resolution (30 m) global digital elevation models over Cameroon (Central Africa) with GPS/leveling ground control points. Int. J. Digit. Earth.

[11] Uuemaa E., Ahi S., Montibeller B., Muru M., Kmoch A. (2020). Vertical accuracy of freely available global digital elevation models (ASTER, AW3D30, MERIT, TanDEM-X, SRTM, and NASADEM). Remote Sens..

[12] Vaka D.S., Kumar V., Rao Y.S., Deo R. Comparison of various DEMs for height accuracy assessment over different terrains of India. Proceedings of the IEEE International Geoscience and Remote Sensing Symposium (IGARSS 2019).

[13] Gdulová K., Marešová J., Moudrý V. (2020). Accuracy assessment of the global TanDEM-X digital elevation model in a mountain environment. Remote Sens. Environ..

[14] Gonzáles-Moradas M.R., Viveen W. (2020). Evaluation of ASTER GDEM2, SRTMv.3.0, ALOS AW3D30 and TanDEM-X DEMs for the Peruvian Andes against highly accurate GNSS ground control points and geomorphological-hydrological metrics. Remote Sens. Environ..

[15] Viel J.A., Rosa K.K., Mendes Júnior C.W. (2020). Avaliação da acurácia vertical dos modelos digitais de elevação SRTM, ALOS World 3D e ASTER GDEM: Um estudo de caso no Vale dos Vinhedos, RS–Brasil. Rev. Bras. Geogr. Fís..

[16] Barbosa V.R.F., Cicerelli R.E., Almeida T., Marotta G.S., Rodrigues S.W.P. (2020). ALOS PRISM (AW3D05 Standard) and Sentinel-1: Evaluation of new sources of digital elevation models. Rev. Bras. Geogr. Fís..

[17] IBGE (1983). Especificações e Normas Gerais para Levantamentos Geodésicos em Território Brasileiro.

[18] IBGE Banco de Dados Geodésicos-BDG-o Que é. https://www.ibge.gov.br/geociencias/informacoes-sobre-posicionamento-geodesico/rede-geodesica/16327-banco-de-dados-geodesicos.html?=&t=o-que-e.

[19] Brazil (1967). Decreto Nº 243, de 28 de Fevereiro de 1967, Fixa as Diretrizes e Bases da Cartografia Brasileira. http://www.planalto.gov.br/ccivil_03/Decreto-Lei/1965-1988/Del0243.htm.

[20] IBGE Sobre a Publicação-Rede Planialtimétrica. https://www.ibge.gov.br/geociencias/informacoes-sobre-posicionamento-geodesico/rede-geodesica/16284-rede-planialtimetrica.html?=&t=sobre.

[21] IBGE Rede Altimétrica-o Que é. https://www.ibge.gov.br/geociencias/informacoes-sobre-posicionamento-geodesico/rede-geodesica/16283-rede-altimetrica.html?=&t=o-que-e.

[22] IBGE (2018). Relatório: Reajustamento da Rede Altimétrica com Números Geopotenciais-REALT-2018.

[23] IBGE Rede Gravimétrica-o Que é. https://www.ibge.gov.br/geociencias/informacoes-sobre-posicionamento-geodesico/rede-geodesica/16286-rede-gravimetrica.html?=&t=o-que-e.

[24] Brazil (1984). Decreto N° 89.817 de 20 de Junho de 1984. Normas Técnicas da Cartografia Nacional. http://www.planalto.gov.br/ccivil_03/decreto/1980-1989/D89817.htm.

[25] DSG (2016). Especificações Técnicas para a Aquisição de Dados Geoespaciais Vetoriais (ET-ADGV).

[26] DSG (2016). Especificação Técnica de Controle de Qualidade de Dados Geoespaciais (ET-CQDG).

[27] Santos A.P., Rodrigues D.D., Santos N.T., Gripp J. (2016). Avaliação da acurácia posicional em dados espaciais utilizando técnicas de estatística espacial: Proposta de método e exemplo utilizando a norma brasileira. Bol. Ciênc. Geod..

[28] Carvalho J.A.B., Silva D.C. (2018). Métodos para avaliação da acurácia posicional altimétrica no Brasil. Rev. Bras. Cart..

[29] IBGE (2019). Biomas e Sistema Costeiro-Marinho do Brasil: Compatível com a Escala 1:250.000, Relatórios Metodológicos.

[30] Myers N., Mittermeier R.A., Mittermeier C.G., Fonseca G.A.B., Jennifer K. (2000). Biodiversity hotspots for conservation priorities. Nature.

[31] Bolfe E.L., Sano E.E., Campos S.K. (2020). Dinâmica Agrícola do Cerrado-Análises e Projeções.

[32] Santana C.A.M., Campos S.K., Marra R., Aragão A.A., Bolfe E.L., Sano E.E., Campos S.K. (2020). Cerrado: Pilar da agricultura brasileira. Dinâmica Agrícola do Cerrado-Análises e Projeções.

[33] Sano E.E., Rosa R., Scaramuzza C.A.M., Adami M., Bolfe E.L. (2019). Land use dynamics in the Brazilian Cerrado in the period from 2002 to 2013. Pesq. Agropec. Bras..

[34] Farr T.G., Kobrick M. (2000). Shuttle Radar Topography Mission produces a wealth of data. Eos Trans. Am. Geophys. Union.

[35] Orlandi A.G., Carvalho Júnior O.A., Guimarães R.F., Bias E.S., Corrêa D.C., Gomes R.A.T. (2019). Vertical accuracy assessment of the processed SRTM data for the brazilian territory. Bol. Ciências Geodésicas.

[36] NASA SRTMGL1 v003-NASA Shuttle Radar Topography Mission Global 1 Arc Second. https://lpdaac.usgs.gov/products/srtmgl1v003/.

[37] NASA (2015). The Shuttle Radar Topography Mission (SRTM) Collection User Guide. https://lpdaac.usgs.gov/sites/default/files/public/measures/docs/NASA_SRTM_V3.pdf.

[38] FGDC Geospatial Positioning Accuracy Standards Part 3: National Standard for Spatial Data Accuracy (FGDC-STD-001-1998). Washington: FGDC. https://www.fgdc.gov/standards/projects/accuracy/part3/chapter3.

[39] Pal M., Yang X., Li J. (2012). Advanced algorithms for land use and cover classification. Advances in Mapping from Remote Sensor Imagery: Techniques and Applications.

[40] Mukul M., Srivastava V., Jade S., Mukul M. (2017). Uncertainties in the Shuttle Radar Topography Mission (SRTM) heights: Insights from the Indian Himalaya and Peninsula. Sci. Rep..

[41] Rodriguez E., Morris C.S., Belz J.E., Chapin E.C., Martin J.M., Daffer W., Hensley S. (2005). An Assessment of the SRTM Topographic Products.

[42] Buckley S.M., Agram P.S., Belz J.E., Crippen E.M. (2020). NASADEM User Guide.

[43] (2020). NASA NASADEM Merged DEM Global 1 Arc Second V001.

[44] Takaku J., Tadono T., Tsutsui K. (2014). Generation of high resolution global DSM from ALOS PRISM. Int. Arch. Photogramm. Remote Sens. Spat. Inf. Sci..

[45] JAXA ALOS Global Digital Surface Model (DSM)-ALOS World 3D-30 m (AW3D30)-Product Format Description, Version 3.1. https://www.eorc.jaxa.jp/ALOS/en/aw3d30/aw3d30v31_product_e_a.pdf.

[46] JAXA ALOS Global Digital Surface Model “ALOS World 3D-30 m (AW3D30). https://www.eorc.jaxa.jp/ALOS/en/aw3d30/index.htm.

[47] Motta L.P. Lapig Msc Giovana Package. Brasília (DF): Github. https://github.com/lmotta/lapig_msc_giovana/tree/main/script/MDE.

[48] Rodrigues T.G., Paradella W.R., Oliveira C.G. (2011). Evaluation of the altimetry from SRTM-3 and planimetry from high-resolution PALSAR FBD data for semi-detailed topographic mapping in the Amazon region. An. Acad. Bras. Ciênc..

[49] Souza M., Ramos A.P.M., Marcato Júnior J. (2019). Analysis of the altimetric accuracy of ALOS AW3D30 digital surface model for Mato Grosso do Sul. Anu. Inst. Geociênc..

[50] GRASS Development Team (2017). Geographic Resources Analysis Support System (GRASS) Software, Version 7.2. Open Source Geospatial Foundation. Electronic Document. http://grass.osgeo.org.

[51] Agrawal R., Mahtab A., Jayaprasad P., Pathan S.K., Ajai Validating SRTM DEM with differential GPS measurements-A case study with different terrains. Proceedings of the Symposium of International Society for Photogrammetry and Remote Sensing.

[52] Shapiro S.S., Wilk M.B. (1965). An analysis of variance test for normality (complete samples). Biometrika.

[53] Anderson T.W., Darling D.A. (1952). Asymptotic theory of certain “goodness of fit” criteria based on stochastic processes. Ann. Math. Stat..

[54] Santos A.P. (2019). Software GeoPEC Versão 3.5.2. Viçosa. http://www.geopec.com.br/p/software-geopec.html.

[55] Mikhail E., Ackerman F. (1976). Observations and Least Squares.

[56] Monico J.F.G., Dal Poz A.P., Galo M., Santos M.C., Oliveira L.C. (2009). Acurácia e precisão: Revendo os conceitos de forma acurada. Bol. Ciênc. Geod..

[57] Hallak R., Pereira Filho A.J. (2011). Metodologia para análise de desempenho de simulações de sistemas convectivos na região metropolitana de São Paulo com o modelo ARPS: Sensibilidade a variações com os esquemas de advecção e assimilação de dados. Rev. Bras. Meteorol..

[58] Willmott C.J. (1981). On the validation of models. Phys. Geogr..

[59] IBGE (2018). Produção Agrícola Municipal (PAM)-Ano Base 2018.

[60] Souza C.M., Shimbo J.Z., Rosa M.R., Parente L.L., Alencar A.A. (2020). Reconstructing three decades of land use and land cover changes in Brazilian biomes with Landsat archive and Earth Engine. Remote Sens..

[61] Cohen J. (1988). Statistical Power Analysis for the Behavioral Sciences.

[62] Rosenthal J.A. (1996). Qualitative descriptors of strength of association and effect size. J. Soc. Serv. Res..

[63] Silva A.S., Santiago O.R.P.L., Silva C.R. (2018). Análise de exatidão entre MDEs: AW3D, SRTM-30 m e projeto SPMGGO50. Geografia.

[64] ASPRS (1990). Accuracy Standards for Large-Scale Maps.

